# Mineralocorticoid Receptor Activation in Vascular Insulin Resistance and Dysfunction

**DOI:** 10.3390/ijms23168954

**Published:** 2022-08-11

**Authors:** Aderonke E. Igbekele, George Jia, Michael A. Hill, James R. Sowers, Guanghong Jia

**Affiliations:** 1 Department of Medicine-Endocrinology and Metabolism, University of Missouri School of Medicine, Columbia, MO 65212, USA; 2 Department of Medical Pharmacology and Physiology, University of Missouri School of Medicine, Columbia, MO 65212, USA; 3 Dalton Cardiovascular Research Center, University of Missouri, Columbia, MO 65212, USA

**Keywords:** mineralocorticoid receptors, aldosterone, insulin resistance, diabetes, vascular dysfunctions

## Abstract

Systemic insulin resistance is characterized by reduced insulin metabolic signaling and glucose intolerance. Mineralocorticoid receptors (MRs), the principal receptors for the hormone aldosterone, play an important role in regulating renal sodium handling and blood pressure. Recent studies suggest that MRs also exist in tissues outside the kidney, including vascular endothelial cells, smooth muscle cells, fibroblasts, perivascular adipose tissue, and immune cells. Risk factors, including excessive salt intake/salt sensitivity, hypertension, and obesity, can lead to the activation of vascular MRs to promote inflammation, oxidative stress, remodeling, and fibrosis, as well as cardiovascular stiffening and microcirculatory impairment. These pathophysiological changes are associated with a diminished ability of insulin to initiate appropriate intracellular signaling events, resulting in a reduced glucose uptake within the microcirculation and related vascular insulin resistance. Therefore, the pharmacological inhibition of MR activation provides a potential therapeutic option for improving vascular function, glucose uptake, and vascular insulin sensitivity. This review highlights recent experimental and clinical data that support the contribution of abnormal MR activation to the development of vascular insulin resistance and dysfunction.

## 1. Introduction

Reduced tissue insulin sensitivity and glucose intolerance are common manifestations of obesity, insulin resistance, and a prelude to type 2 diabetes [1]. Human and animal studies have demonstrated that elevated aldosterone levels and mineralocorticoid receptor (MR) activation are related to insulin resistance development and progression [1,2,3]. In this regard, MRs are members of the steroid receptor family which are activated by aldosterone. MRs have been well-characterized in renal epithelial cells where their activation induces the expression of proteins that regulate ion and water transport, sodium uptake, and consequent increases in extracellular fluid volume and blood pressure [1,2,3]. Recent data demonstrate that MRs also exist in cardiovascular tissue and play important roles in the development of cardiovascular disease (CVD) and the related cardiometabolic syndrome [4,5]. The latter is a combination of metabolic disorders characterized by insulin resistance, impaired glucose tolerance, obesity, dyslipidemia, hypertension, and CVD [1,6]. Importantly, both clinical and animal studies support the notion that elevated aldosterone levels are a predictor for risk of coronary heart disease, stroke, and hypertension, while MR antagonists diminish CVD and cardiometabolic syndrome mortality [7,8,9,10]. Related to this, enhanced MR activation inhibits insulin secretion and insulin metabolic signaling [1]. Activated MRs also decrease glucose uptake and promote inflammation, excessive oxidative stress, and lipid disorders [1]. These abnormalities result in systemic and tissue insulin resistance [1]. While much is known about the role of MRs in CVD and the related cardiometabolic syndrome, the direct contribution of vascular MRs to the development of vascular insulin resistance and dysfunction is less well understood. In the present review, we cover the potential roles and mechanisms by which MR activation impairs vascular insulin metabolic signaling and subsequently impacts the development of excessive vascular stiffening and hypertension. We also discuss therapeutic approaches for the inhibition of vascular MR activation and consequential improvements in vascular insulin sensitivity and function.

## 2. Aldosterone and MRs

**Figure 1:**
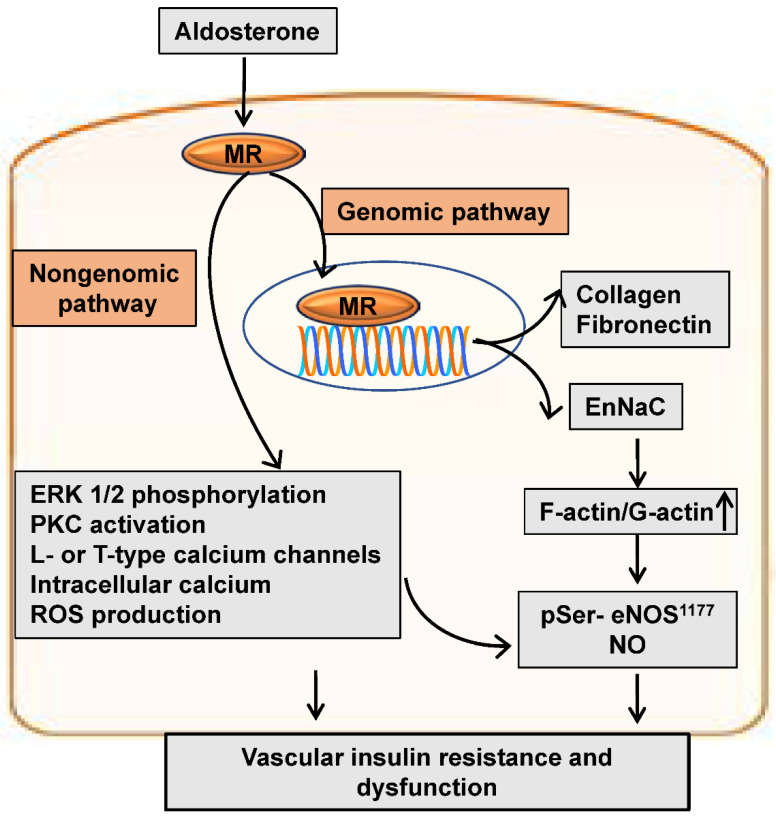
Enhanced mineralocorticoid receptor signaling induces vascular insulin resistance and dysfunction through genomic and nongenomic pathways. Arrow represents a process in vascular insulin resistance. MR: Mineralocorticoid receptor; ERK 1/2: Extracellular receptor kinase 1/2; PKC: Protein kinase C; ROS: Reactive oxygen species; NO: Nitric oxide; eNOS: Endothelial NO synthase.

Aldosterone is synthesized in the zona glomerulosa of the adrenal gland in response to high levels of potassium, angiotensin II, and adrenocorticotropin stimulation [11,12]. Aldosterone is also produced in adipose tissue via aldosterone synthase and aldosterone secretagogues resident within the fat [11,12]. Elevated aldosterone levels directly induce MR expression and activation. Recent data indicate that MRs can be activated in normal- or even low-aldosterone states through a ligand-independent pathway [13]. It was revealed that excess reactive oxygen species (ROS) can activate Rac Family Small GTPase 1 to induce an increase in both MR expression and activation. Thus, excess ROS production, as a modulator of mineralocorticoid receptor activity [14] and known to be elevated in cardiometabolic disease, conceivably plays an important role in the pathogenesis of vascular insulin resistance and dysfunction. Aldosterone directly binds cytosolic MRs, which subsequently translocate to the nucleus, where it binds to specific regions of the DNA containing MR response elements [15]. The binding of the aldosterone–MR receptor complex to these DNA-responsive elements leads to downstream gene transcription and translation, such as for collagen and fibronectin (Figure 1) [15]. Our recent data also suggest that epithelial sodium channels (Na^+^) in ECs (EnNaC) are related to activated MRs-induced genomic pathways [16,17]. To this point, increased EnNaC expression and activation results in the increased Na^+^ influx and polymerization of G-actin to F-actin, which leads to reduced vascular endothelial nitric oxide (NO) synthase (eNOS) activity, NO production, impaired microcirculation, and related glucose intolerance (Figure 1) [16,17]. Aldosterone also activates rapid, non-genomic pathways, including extracellular receptor kinase 1/2 (ERK 1/2), protein kinase C (PKC), and Rho kinase, to indirectly induce increases in nicotinamide adenine dinucleotide phosphate (NADPH) oxidase activation, ROS generation, and mitochondrial dysfunction (Figure 1) [18]. For instance, enhanced nongenomic aldosterone signaling in vascular smooth muscle cells (VSMCs) has been demonstrated to lead to vasoconstriction by increases in ERK 1/2 phosphorylation, PKC activation, L- or T-type calcium channels, elevated intracellular calcium, and ROS production, which indirectly aggravates pathological MR effects in vascular insulin resistance and dysfunction [19]. In endothelial cells (ECs), chronic aldosterone stimulation can inactivate eNOS and NO production, which facilitates pathological MR effects toward vascular dysfunction (Figure 1) [19].

Genetic, epigenetic, and posttranslational modifications affect MR expression and activity. For instance, MRI180V (rs5522) and the MR-2G/C (rs2070951) are two single nucleotide polymorphisms that regulate the transactivational capacity of the MR gene in response to dexamethasone or cortisol stimulation [20]. MicroRNA-124 and microRNA-135a have also been found to regulate MR expression via epigenetic regulation [21]. Posttranscriptional modifications, including oxidation, phosphorylation, sumoylation, and ubiquitylation, are involved in MR expression and activation. In this regard, the Rac Family Small GTPase 1 is found to directly activate MRs leading to nuclear translocation, as evidenced by increased oxidative stress and ROS generation [22]. High glucose or high sodium also stimulate MR transcriptional activity via protein kinase C signaling and oxidative stress [22]. Other receptors, including the epidermal growth factor receptor, glucocorticoid receptor, and estrogen receptor α, also play important roles in the regulation of MR expression and activity [22].

Previous studies have also demonstrated that MR mutations are associated with the regulation of salt sensitivity, blood pressure, and stress responses [23]. Inactivating mutations of MRs are responsible for autosomal dominant disorders and some sporadic cases of pseudohypoaldosteronism type I (PHA1), which is characterized by neonatal renal salt wasting with dehydration, hypotension, and hyperkaliemia, despite elevated aldosterone levels [24]. One study has identified heterozygous mutations in one sporadic and four dominant kindreds by screening the MR gene for variants [25]. MR mutations include two frameshift mutations (one a de novo mutation), two premature termination codons, and one splice donor mutation [25]. Indeed, there are more than fifty different MR mutations that have been found and all disease-related mutations are located in all exons of the NR3C2 gene that codes the functional domains of the MRs [23,26]. Recently, a gain-of-function mutation of the MRs was found to be involved in a familial form of inherited mineralocorticoid hypertension exacerbated by pregnancy [27]. Biochemical testing of the mutated human MR L810 reveals an activation of the MRs even in the absence of aldosterone stimulation [27]. Despite these observations, little data are currently available to directly support whether MR mutations are associated with vascular insulin resistance and diabetes.

## 3. Evidence Implicating MR in Insulin Resistance and Cardiometabolic Disorders 

The Framingham Heart Study has highlighted the coexistence of insulin resistance, diabetes, and cardiometabolic syndrome, specifically reporting that 19% of patients with heart failure have insulin resistance and diabetes and that the risk of heart failure increases by 2–8-fold in diabetic individuals [28,29]. To this point, a 1% increase in glycated hemoglobin is related to an 8% increase in risk of heart failure, independent of body mass index, high blood pressure, and age [28,29]. Conversely, a 1% reduction in glycated hemoglobin is associated with a 16% reduced risk of developing heart failure and worsening clinical outcomes [28,29]. The Framingham Offspring Study further demonstrated that there was a correlation between elevated plasma aldosterone levels and the temporal appearance of hypertension, cardiac dysfunction, and other cardiometabolic syndrome markers within this study group [30]. Consistent with the clinical evidence, preclinical studies also support that elevated aldosterone levels and increased MR expression are related to the pathophysiology of insulin resistance and the cardiometabolic syndrome in experimental models. Our studies and that of others have found that enhanced MR signaling plays a key role in obesogenic diet-induced increases in arterial stiffening, hypertension, and renal and cardiac diastolic dysfunction [31]. Conversely, inhibition of MRs with FAD286 (an aldosterone synthase inhibitor) and MR antagonists (spironolactone and eplerenone) inhibit excessive oxidative stress and proinflammatory responses, improve glucose tolerance and insulin sensitivity, and prevent the diet-induced development of cardiometabolic syndrome [1]. Several mechanisms contributing to aldosterone/MR activation-induced glucose intolerance and insulin resistance have been indicated, including impaired pancreatic beta cell function and related increases in hepatic gluconeogenesis. Thus, recent studies have shown that elevated endogenous aldosterone levels repress glucose-stimulated insulin secretion through an MR-independent mechanism [32]. Enhanced MR signaling also induces an impairment in hepatic insulin metabolic signaling which contributes to hepatic glucose intolerance and increased hepatic gluconeogenesis [33]. Another study showed that the MR antagonist, spironolactone, promoted white adipose tissue browning and hepatic glucose transporter type 4 expression, suggesting that MR signaling mediates diet-induced hepatic steatosis, the dysregulation of adipose tissue browning, and systemic and tissue insulin resistance [33]. 

## 4. Role of Vascular MRs 

**Figure 2:**
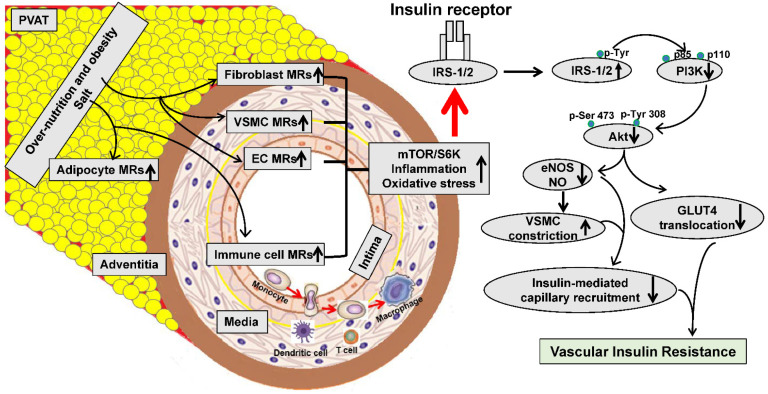
Proposed mechanisms for the inappropriate mineralocorticoid receptor activation in the pathogenesis of vascular insulin resistance. Arrow represents a process in vascular insulin resistance. MR: Mineralocorticoid receptor; PVAT: Perivascular adipose tissue; EC: Endothelial cell; VSMC: Vascular smooth muscle cell; mTOR: Rapamycin (mTOR); S6K1: Ribosomal S6 kinase; IRS-1: Insulin receptor substrate 1; PI3K: Phosphoinositide 3-kinases; Akt: Protein kinase B; GLUT4: Glucose transporter 4; NO: Nitric oxide; eNOS: Endothelial NO synthase.

The blood vessel wall is principally composed of intima vascular ECs, media VSMCs, adventitial fibroblasts, perivascular adipose tissue, and immune cells (Figure 2). While the role of the systemic and tissue activation of MRs in CVD is well-established, its exact relationship with vascular insulin resistance and dysfunction represents a relatively new area of investigation. Recent data indicate that MRs exist in various cell types of the vascular wall, in both animals and human vascular tissues, that regulate vascular physiology and contribute to pathological states [1,2,3] (Figure 2).

## 5. MR in Vascular ECs 

Our recent and other studies support an important role for endothelial MRs (ECMRs) in the development of endothelial damage that underlies impaired endothelial dependent vasodilation. For instance, in a diet-induced obesity mouse model, impaired endothelial-dependent (NO-mediated) vasodilation is prevented by either MR inhibition with spironolactone [31] or ECMR gene knockout (KO) [17]. Other studies have also shown that ECMR KO prevents the angiotensin-II-induced impairment of vascular relaxation and the development of hypertension [34]. ECMRs are also involved in diet/hypertension-induced cardiac fibrosis and heart failure [35,36]. Endothelial oxidative stress, inflammation, and reduced NO production are involved in these pathophysiological processes [35]. One of the important characteristics in ECMR activation is that enhanced ECMR signaling contributes to vascular leukocyte adhesion, infiltration, and related local inflammatory responses [37]. To this point, activated MRs in ECs induce increases in EC adhesion molecules, including selectins, the vascular cell adhesion molecule (VCAM)-1, and the intracellular adhesion molecule (ICAM)-1, which promote leukocyte–endothelial adhesion and facilitate trans-endothelial infiltration into the vasculature [37]. This effect of enhanced endothelial MR activation on endothelial permeability could represent a mechanism by which the endothelial MR specifically promotes immune cell transmigration and infiltration to the subendothelial layer contributing to early vascular dysfunction. Interestingly, ECMR sex-dependently regulates the expression of selectin, ICAM-1, and VCAM-1 for leukocyte recruitment. For instance, when compared to female littermates, tumor necrosis factor alpha (TNFα)-induced mesenteric vasculature expression of E-selectin is lower than that of males and is not further affected by the gene deletion of ECMR [19]. Moreover, the expression of E-selectin is lower in females than in males. Further, E-selectin has been shown to be negatively regulated by estrogen [19]. 

## 6. MR in VSMCs

MRs are also expressed in VSMCs. In response to enhanced mechanical or physiological stress, VSMC MRs are activated to induce VSMC hypertrophy, migration, proliferation, and vascular remodeling, as well as overt vascular fibrosis [19,38]. For instance, aldosterone has been shown to enhance the VSMC expression of collagen I and III, bone morphogenetic protein 2, and alkaline phosphate [19,38], which are associated with vascular remodeling, fibrosis, and atherosclerosis. These detrimental vascular effects of activated VSMC MRs occur synergistically with the presence of EC dysfunction but independent of changes in blood pressure [39]. Recently, a specific VSMC MR KO mouse model was developed, allowing the direct study of the actions of VSMC-MRs in vascular dysfunction. In this regard, MR deletion in VSMCs has been shown to inhibit thromboxane, angiotensin II, and calcium channel agonist-induced increases in vascular contractile responses and vascular tone, while also decreasing the contractile response in the arteries of aged mice [40]. The VSMC MR gene KO further prevented angiotensin II-induced increases in vascular superoxide production and systemic blood pressure [40]. Signaling pathways, including those involving Rho-kinase and placental growth factor (PGF) signaling through vascular endothelial growth factor type 1 receptors, were implicated as candidate mechanisms underlying the activated VSMC MR-induced vascular dysfunction. For instance, aldosterone increases Rho-kinase activity, stress fiber formation, migration, and the proliferation of in vitro VSMC in an MR-dependent manner [41]. c-Src phosphorylation mediates aldosterone-induced activation of the Rho-kinase pathway [41]. Meanwhile, aldosterone also increases PGF expression, which is identified as a novel target of MR signaling in VSMCs with enhanced expression in the setting of endothelial injury [42]. The gene deletion of PGF inhibits aldosterone-induced VSMC proliferation, extracellular matrix (ECM) deposition, and medial vessel thickening [43]. These data indicate that VSMC-specific MR signaling contributes to vascular dysfunction. 

## 7. MR in Fibroblast and ECM

MRs also exist in fibroblasts, and their activation facilitates ECM remodeling and vascular fibrosis [44]. Data derived from studies of rat cardiac myofibroblasts showed that aldosterone-induced fibroblast proliferation was mediated through the activation of the Kirsten Ras/mitogen-activated protein kinase-1/2 signaling pathway [45]. Aldosterone increased the expression of galectin-3 in human cardiac fibroblasts, which, in turn, promoted profibrotic marker expression and inflammation [46]. Aldosterone has also been shown to induce plasminogen activator inhibitor type-1 (PAI-1) expression via MR activation in multiple tissues, including the heart, kidney, and aorta [47]. Related to this, activated PAI-1 directly inhibits urokinase and tissue plasminogen activator and promotes vascular fibrosis [48]. Increased PAI-1 expression promotes ECM accumulation, vascular fibrosis, and remodeling by inhibiting plasmin activation [48]. Meanwhile, excessive aldosterone increases vascular fibrosis, in part, by increasing growth factor beta 1 (TGF-β1)/Smad signaling [35]. To this point, TGF-β1 is expressed in all vascular cells, including VSMCs and fibroblasts. Enhanced TGF-β1 expression and activation promotes the synthesis and accumulation of ECM proteins, including collagen and elastin [49], which are regulated by matrix metalloproteinases. Activated matrix metalloproteinases degrade elastin and collagen and further promote vascular remodeling and fibrosis. Moreover, increased activity of tissue transglutaminase (TG2), an ECM scaffold protein and crosslinking enzyme, promotes the crosslinking of collagen and the related vascular remodeling and fibrosis [49]. TG2 also activates TGF-β1/Smad signaling to induce collagen synthesis and vascular fibrosis [49].

## 8. MRs in Immune Cells 

Functional MRs are widely expressed in a variety of immune cells, including macrophages, T lymphocytes, and dendritic cells. The in vitro treatment of macrophages with aldosterone results in MR activation and further promotes the expression of the classical M1 activation markers, namely TNFα, monocyte chemoattractant protein-1 (MCP1), and interleukin (IL)-12, in mouse peritoneal macrophages [50,51]. Similarly, aldosterone-induced MR activation potentiates the lipopolysaccharide (LPS)-induced release of pro-inflammatory cytokines, TNFα and IL-6, in an MR-dependent manner in an immortalized mouse microglial cell line [52]. The transcription factor nuclear factor κ-light-chain enhancer of activated B cells (NF-κB) mediates the expression and release of these cytokines in a variety of immune cells [53]. The MR activation in myeloid cells has also been implicated in vascular remodeling and dysfunction. Of direct relevance to the vasculature, the depletion of MRs in myeloid cells prevents angiotensin-II-induced macrophage infiltration and M1 polarization in arterial tissues [54]. Further, an MR deficiency in macrophages reduces the expression of M1 macrophage activation markers, whereas the expression of alternative activation/M2 polarization markers significantly increases, suggesting that MR activation primarily favors the polarization of M1 over M2 macrophages [51]. MRs also exist in T and B lymphocytes [50]. In vivo rodent studies have shown that deoxycorticosterone acetate (DOCA) treatment increases the polarization of T cells toward the Th1/Th17 phenotype through MRs [55]. The role of DOCA on Th/Th17 polarization was prevented by eplerenone and spironolactone. Meanwhile, DOCA treatment also decreases Treg cell abundance in an MR-dependent manner [56], suggesting a role of MRs in increasing the activation of T lymphocytes. Indeed, MRs also exist in B lymphocytes, with one study showing that the expression of MRs is equivalent in T and B lymphocytes [57]. However, the role of MRs in B lymphocytes remains unclear. It has been shown that the activated MR signaling on dendritic cells promotes Th17 lymphocyte-mediated immunity, and an MR antagonist in hypertensive rats treated with DOCA-salt represses the Th17 response in heart and peripheral blood by modulating a regulatory T cell [58]. Moreover, aldosterone induces MR activation to increase the expression of IL-6 and TGFβ in dendritic cells, which further contributes to the effects of aldosterone on T lymphocyte Th1/Th17 polarization [50]. 

## 9. MRs in Perivascular Adipose Tissue (PVAT)

Aldosterone, although predominantly synthesized and secreted in the adrenocortical cells of the zona glomerulosa within the adrenal cortex, is also produced in adipocytes, including in PVAT [1,6]. Adipocyte tissue can be classified into white, brown, and beige adipocyte tissues. While white adipocyte tissue is the primary site for fat expansion and energy storage, the excess expansion of white adipocyte tissue, especially that in visceral fat, is associated with insulin resistance, type 2 diabetes, and metabolic syndrome [1]. However, brown adipocyte tissue is regarded to have a beneficial role in the production of heat energy through non-shivering thermogenesis [6,33]. Upon being activated by aldosterone, activated MRs promote adipogenesis through the transcription factors in the peroxisome proliferator-activated receptors (PPARs) and CCAAT-enhancer-binding proteins (C/EBPs) [1]. Studies have also reported that adipose tissue MR-mediated activation of the mammalian target of the rapamycin (mTOR)/ribosomal S6 kinase (S6K1) signaling pathway further activates PPARs and C/EBP signaling to promote adipogenesis in diet-induced obesity [1]. Meanwhile, activated MRs also regulate adipose differentiation. Thus, an earlier study has shown that increased MR promoter activity in a transgenic mice model, expressing the SV40 large T antigen, enhanced the transcriptional MRs in the adipocytes and induced liposarcoma development [1]. Our recent data further showed that reduced MR activity promoted white adipose tissue browning, as evidenced by the increased expression of the uncoupling protein 1, deiodinase 2, β3 adrenergic receptor, and PR domain-containing 16 in fat tissues [33]. Importantly, MR activation in fat tissue induces oxidative stress and inflammation that further impair the neighbor cell and tissue physiological function, leading to vascular insulin resistance and dysfunction.

## 10. MR Activation and Vascular Insulin Resistance

Insulin resistance is characterized by a diminished ability of insulin to initiate insulin metabolic signaling and glucose uptake [1]. The primary targets of insulin include skeletal muscle and liver and fat tissues. However, insulin receptors and insulin metabolic signaling are also present in vascular cells. Impaired insulin signaling in vascular tissues inhibits glucose uptake and induces vascular insulin resistance (Figure 2). 

Experimental studies indicate the fact that excessive MR activation directly impairs insulin metabolic signaling and induces vascular stiffness [17,31,33]. Related to this, our data suggest that enhanced MRs activate the mTOR/S6K1 signaling pathway, which promotes vascular insulin resistance through the increased serine phosphorylation of the critical insulin signaling/docking molecule insulin receptor substrate 1 (IRS-1) [1] (Figure 2). This serine phosphorylation then impairs the IRS-1 tyrosine phosphorylation, phosphoinositide 3-kinases (PI3K) engagement, and protein kinase B (Akt) phosphorylation/activation, as well as the downstream translocation of glucose transporter 4 (GLUT4) to the cell membrane and glucose uptake [1]. On the one hand, reduced insulin metabolic signaling via PI3K/Akt inhibits the eNOS activation and NO production, leading to increased VSMC calcium sensitization and reduced vasodilation [1]. On the other hand, the hyperinsulinemia associated with vascular insulin resistance increases the release of endothelin-1 (ET-1) via a mitogen-activated protein kinase (MAPK)-dependent signaling pathway [1]. Thus, endothelial insulin resistance is accompanied by a reduced PI3K-NO pathway and heightened MAPK-ET-1 pathway. Meanwhile, reduced eNOS activity and NO in vascular insulin resistance promote the phosphorylation of myosin light-chain kinase (MLCK), Ca^2+^ MLCK sensitization, leading to decreased VSMC relaxation, vascular stiffening, and impaired microcirculation [1]. (Figure 2).

Enhanced vascular MR signaling induces vascular insulin resistance by increased inflammation and oxidative stress [6]. On the one hand, activated MRs increase the activation of proinflammatory T helper (Th) cells and macrophage M1 polarization [17,31]. On the other hand, enhanced MRs increase the expression of proinflammatory adipokines, including TNFα, IL-6, MCP-1, and toll-like receptor 4 in adipose tissue [6,33]. These proinflammatory responses activate NF-κB and c-Jun NH_2_-terminal kinase (JNK) to directly promote the serine phosphorylation of IRS-1 and induce impaired insulin metabolic signaling [1]. Meanwhile, excessive oxidative stress, induced by vascular MRs, activates redox-sensitive serine kinases and induces the serine phosphorylation of IRS-1, resulting in impaired vascular insulin metabolic signaling and insulin resistance [1]. Consistent with previous studies, our recent data also found that the inhibition of MRs with spironolactone decreased the expression of TNFα, MCP-1, IL-6, CD68, and CD11c, as well as arterial 3-nitrotyrosine (a biomarker of oxidative stress) with parallel increases in vascular insulin sensitivity [31]. These data suggest that MR activation promotes vascular proinflammatory responses and related vascular insulin resistance and dysfunction.

## 11. Role of Vascular MR Activation and Insulin Resistance in Arterial Stiffening and Hypertension: Potential Therapeutic Strategies 

Vascular MR activation and insulin resistance independently or synergistically contribute to arterial stiffening and hypertension. For instance, a prospective study of 566 individuals with uncontrolled hypertension demonstrated that MR inhibition with spironolactone alleviates arterial stiffening in parallel with reduced blood pressure [59]. Another clinical study using a biracial population of 4701 men and women aged 45–64 found that patients with non-insulin-dependent diabetes mellitus or borderline abnormal glucose intolerance had stiffer arteries than individuals with normal glucose tolerance, and the reduced elasticity was independent of artery wall thickness [60]. Our understanding of insulin resistance-induced hypertension is currently evolving. Early data from 19 patients with essential hypertension but without evidence of diabetes demonstrated that plasma insulin levels were higher in these patients than in the control group [61]. Interestingly, about 50% of the participants with hypertension exhibited insulin resistance [62]. The Framingham Offspring Study of 1933 normotensive patients investigated the relationship between insulin resistance and the 4-year incidence of hypertension and its progression. This analysis demonstrated that insulin resistance was positively related to hypertension after adjusting an increased body mass index [63]. These data suggest that interactions between activated vascular MR signaling and insulin resistance promote arterial stiffening and hypertension.

Primary adrenal insufficiency is characterized by the insufficient secretion of glucocorticoids, mineralocorticoids, and androgens [64]. Secondary adrenal insufficiency usually occurs because of exogenous steroid administration, resulting in the suppression of adrenocorticotropic hormone synthesis [64]. Patients with primary adrenal insufficiency exhibit double the mortality risk from CVD, including hypotension, arrhythmias, and heart failure, and thus these individuals are required to have hormonal replacement treatment [64]. However, both glucocorticoid and mineralocorticoid replacement therapies increase the risk of CVD. One study showed that corticosteroid therapy mismatches increased mortality by 1.5–2-fold, reducing life expectancy by 11 years in males and by 3 years in females [65]. Cushing’s syndrome is an endocrine disorder induced by increased endogenous cortisol secretion or the exogenous administration of glucocorticoids [66]. Cushing’s syndrome is not only related to increased CVD risk, such as atherosclerosis, coronary heart disease, and heart failure, but is also associated with metabolic syndromes, including obesity, glucose intolerance, insulin resistance, and diabetes [66]. It is accepted that Cushing’s syndrome can induce EC and VSMC dysfunction through MR signaling and lead to increased vascular inflammation, atherosclerosis, and vascular insulin resistance [66]. 

MR antagonists, eplerenone and spironolactone, are currently approved for clinical patients. MR antagonists have a conclusive beneficial role in reducing blood pressure, especially in resistant hypertension [49]. Recent preclinical data also support that spironolactone and eplerenone improve glucose metabolism and prevent insulin resistance [1]. However, data from the Candesartan in Heart Failure Assessment of Reduction in Mortality and Morbidity (CHARM) clinical study indicated that MR antagonists may be related to an increased risk for the development of diabetes [67]. Therefore, the impact of MR antagonists in patients with glucose intolerance, insulin resistance, and diabetes is not clear yet. Traditional MR antagonists, including eplerenone and spironolactone, are effective treatments for heart failure and resistant hypertension but have adverse effects, such as hyperkalemia as well as being antiandrogenic and progestogenic. Recent clinical studies evaluating the non-steroidal MR antagonists, including finerenone, in clinical patients with hypertension and heart failure have provided encouraging results [68]. Finerenone is also found to activate brown adipose tissue and thus prevented high fat diet–induced insulin resistance and the cardiometabolic syndrome [69]. Clinical studies further suggest that finerenone has a high affinity and specificity for the MR and is a potential new drug in cardiometabolic syndrome therapy [3,10]. 

## 12. Conclusions

MRs exist in vascular tissue, including ECs, VSMCs, ECM fibroblasts, and vascular immune cells, as well as PVAT. Excessive salt consumption/salt sensitivity, hypertension, and obesity are CVD risk factors associated with the excessive activation of vascular MR signaling that causes vascular inflammation, oxidative stress, remodeling, and fibrosis, leading to vascular insulin resistance, vascular stiffening, impaired vascular relaxation, and hypertension. Of note, the MRs have three ligands: aldosterone, cortisol, and progesterone. While ECs and VSMCs express the enzyme 11-beta hydroxysteroid dehydrogenase 2 (11βHSD2), allowing aldosterone to selectively activate MRs by inactivating cortisol, vascular fibroblasts, immune cells, and PVAT do not express 11βHSD2, and thus the main ligand for MRs is cortisol in these cells [70]. Indeed, aldosterone and glucocorticoids have an equal affinity with MRs, so it is difficult to distinguish the events induced by activated MRs and glucocorticoid receptors [70]. The affinities of glucocorticoids for MRs are ten-fold higher than those for glucocorticoid receptors [70]. However, glucocorticoid receptors have a lower affinity to both glucocorticoids and aldosterone. Therefore, it is also difficult to differentiate the role of glucocorticoids on MR expression, activation, and related vascular insulin resistance as glucocorticoids have the capacity to activate both MR and glucocorticoid receptors. Further studies are warranted to understand the role and mechanism of MR activation in vascular insulin resistance and dysfunction.
